# At the dark end of the rainbow: data gaps in tattoo toxicology

**DOI:** 10.1007/s00204-016-1740-9

**Published:** 2016-05-11

**Authors:** Ines Schreiver, Andreas Luch

**Affiliations:** Department of Chemical and Product Safety, German Federal Institute for Risk Assessment (BfR), Berlin, Germany

Toxicology and consumer safety have significantly improved since the beginning of the last century. In current times, thorough testing of goods is being premised in our society. Yet this trust might be questioned with regard to the colorful inks used in tattooing or permanent makeup.

The revival of tattooing in Europe during the last century arose from sailors traveling the world and linking the image of “the daring” to tattooed individuals (Laux et al. 2016). In addition, apparent risks from poor tattoo equipment and adventurous inks mixed from ashes, red bricks, plant leaves and varnishes disappeared as a result of the professionalization of the tattoo industry. A closer look on the history of legislation in this field reveals the remaining pain points of tattoo safety though. Due to the lack of toxicological data, currently safety assessment is mainly deduced from cosmetic ingredients. As a consequence, tattoo legislation in Europe is still based on an exposure scenario placing the product on top of the skin, rather than right into the middle of living tissue beneath the epidermal skin barrier. Certainly, the presence of blood vessels and neurons considerably affects both the bioavailability of pigments, auxiliary ingredients and contaminants on the one hand, as well as their tissue and cellular interactions on the other. Although the limited regulation of tattoo inks is now recognized for some time, the putatively low prevalence of severe side effects has rather led to a “show-me-the-dead-bodies” mind set in the general public and of healthcare officials alike.

Indeed, microbiological outbreaks in Europe are rare (Drage et al. 2010). Further, although still occasionally occurring they seem manageable by improved hygienic standards. On the other hand, allergic side effects may have a major impact on the health of consumers (Kluger 2015; Brady et al. 2015; Wenzel et al. 2013). Since the ink is being administered into deeper layers of the skin, the obvious and only effective therapeutic counteraction, an immediate removal of the respective allergen remains unfeasible in the case of tattooed skin. While consumers affected by cosmetics can easily remove and avoid the allergen, tattooed individuals are unable to terminate the exposure right after the onset of symptoms (redness, swelling, itching, inflammation, etc.). For sure, this difference triggers one of the major health concerns and actually points to an inept equalization of tattoo inks with cosmetics. Even the method of laser-mediated pigment removal is usually to be refused when allergic reactions become apparent since the procedure might further mobilize ink particles via fragmentation. In some cases, it even may promote the formation of the actual allergenic species (Bernstein 2007). As a last resort and *ultima ratio*, in the case of severe allergic side effects only invasive surgery including transplantation seems applicable if anti-inflammatory treatment fails to limit the damage and to calm down the reaction.

The mostly feared and discussed risk factor for tattooing is the supposably carcinogenic potential of the inks applied. However, available studies that evaluated the association between skin (and other types of) cancer and the presence of tattoos failed to reveal any connection due to an extremely low number of cases (Kluger and Koljonen 2012). As a great disadvantage, past cohort and case–control studies did not include tattooing as possible risk factor although theoretically suited to link or to reject any connection between tattoos and cancer. Irrespective of the lack of epidemiological data, genotoxic and carcinogenic primary aromatic amines (pAAs) or polycyclic aromatic hydrocarbons (PAHs) are frequently detected as impurities of tattoo inks. Due to their bioavailability, they can travel across the body and its organs and tissues, including those more prone to be affected by these compounds (i.e., target tissues). For sure, the analytical detection of genotoxic carcinogens in tattoo ink formulations that are supposed to be injected into skin tissue well supplied with blood, this fact alone might already trigger serious concerns.

Even when produced in a highly pure manner, tattoo pigments still bare the risk of getting fragmented or converted by ultraviolet and visible light, laser irradiation and/or cellular metabolism. To adequately address possible long-term (chronic) health effects, ingredients of tattoo inks in their original form as well as descendants abiotically or biotically formed over time within the human body need to be considered and seriously assessed (Laux et al. 2016). Already today, many of the hazardous substances present in tattoo inks or originating over time from certain ingredients are toxicologically characterized (e.g., pAAs, PAHs). Besides hazard identification, the evaluation of compound-specific biokinetics and internal target tissue exposure levels are of utmost importance to reliably assess the long-term risks associated with this increasingly popular form of body modification.

In the present issue of “Archives of Toxicology,” we propose to employ pyrolysis-mediated fragmentation followed by gas chromatographic separation and mass spectrometric detection as highly sensitive method to identify hazardous substances that are likely to emerge through tattoo pigment decomposition during sunlight exposure or laser irradiation. The types of pigments applied have become famous for their high color brilliance, and due to their low purchasing costs impressively bright and colorful skin modifications become more common and pervasively visible in the mainstream. The data collected in our study reveal that most organic pigments used in tattoo inks are thermically cleavable to yield potentially harmful substances. In particular, azo pigments might be decomposed into pAAs that can make up to 20 % of the total peak area found in the corresponding pyrograms. The formation of genotoxic and carcinogenic pAAs has already been reported after sunlight and laser irradiation of azo pigments (Engel et al. 2010). Compared to azo compounds, other more lightfast pigments like quinacridones, diketopyrrolo-pyrrols and phthalocyanines revealed less decomposition products when applying similar temperatures. Nonetheless, we found that essentially all organic tattoo pigments can be cleaved into toxins such as benzene, cyanides and others, upon administration of sufficient amounts of thermal energy. On the other side, such heavy decomposition is as yet not described in the literature to occur upon sunlight exposure. Yet own work has shown laser irradiation being capable of cleaving copper phthalocyanine blue pigments into the same pattern of fragments as found in pyrolysis at 800 °C (Schreiver et al. 2015). With this, we proved artificial pyrolysis as suitable method to predict the identity of descendants that emerge upon both thermal and photochemical pigment cleavage.

Taking into account that—at average—about 2.5 mg pigments will be deposited inside the skin per square centimeter (Engel et al. 2008) while creating an upper arm tattoo of 400 cm^2^, an individual body load of 1 g of particles can be calculated (Fig. 1). Highly tattooed people may even carry up to 40 g of pigment within their body. Size, color and particle density of the respective tattoo will thus determine the amount of possible harmful substances as well as the portion of hazardous decomposition products that chronically affect skin and lymph nodes (Anderson et al. 1996). Future investigations are urgently required to assess the quantities of the proposed compounds evolving from the pigments upon laser treatment or during chronic sunlight exposure. These data can then be used to describe possible worst-case scenarios and to reliably assess the health risks that might be associated therewith. Certainly, the great variety of pigments and other ingredients used in ink formulations will make it rather a long way to go to clear the dark end of the tattoo rainbow—eventually.

**Fig. 1:**
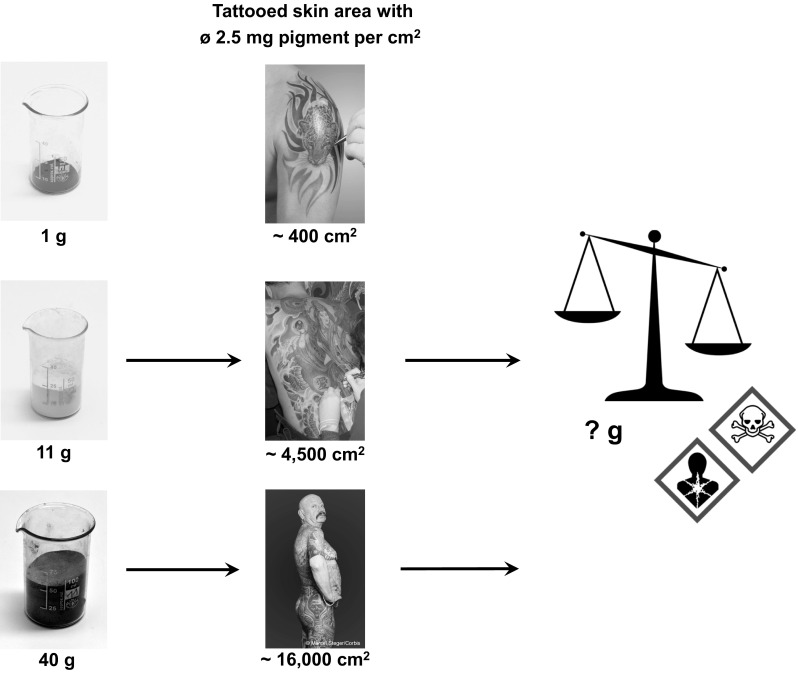
Average pigment deposition per tattoo surface area. Quantities of possible hazardous compounds deriving from tattoo pigments by different decomposition routes (thermal, sunlight, laser) are still unknown. *Picture* at the *bottom* of the *middle column*: courtesy of Marcel Steger/Corbis (©Marcel Steger/Corbis)

## References

[CR1] Anderson LL, Cardone JS, McCollough ML, Grabski WJ (1996). Tattoo pigment mimicking metastatic malignant melanoma. Dermatol Surg.

[CR2] Bernstein EF (2007). Laser tattoo removal. Sem Plastic Surg.

[CR3] Brady BG, Gold H, Leger EA, Leger MC (2015). Self-reported adverse tattoo reactions: a New York City Central Park study. Contact Dermat.

[CR4] Drage LA, Ecker PM, Orenstein R, Phillips PK, Edson RS (2010). An outbreak of *Mycobacterium chelonae* infections in tattoos. J Am Acad Dermatol.

[CR5] Engel E, Santarelli F, Vasold R, Maisch T, Ulrich H, Prantl L, König B, Landthaler M, Bäumler W (2008). Modern tattoos cause high concentrations of hazardous pigments in skin. Contact Dermat.

[CR6] Engel E, Vasold R, Santarelli F, Maisch T, Gopee NV, Howard PC, Landthaler M, Bäumler W (2010). Tattooing of skin results in transportation and light-induced decomposition of tattoo pigments—a first quantification in vivo using a mouse model. Exp Dermatol.

[CR7] Kluger N (2015). Self-reported tattoo reactions in a cohort of 448 French tattooists. Int J Dermatol.

[CR8] Kluger N, Koljonen V (2012). Tattoos, inks, and cancer. Lancet Oncol.

[CR9] Laux P, Tralau T, Tentschert J, Blume A, Al Dahouk S, Bäumler W, Bernstein E, Bocca B, Alimonti A, Colebrook H, de Cuyper C, Dähne L, Hauri U, Howard PC, Janssen P, Katz L, Klitzman B, Kluger N, Krutak L, Platzek T, Scott-Lang V, Serup J, Teubner W, Schreiver I, Wilkniß E, Luch A (2016). A medical-toxicological view of tattooing. Lancet.

[CR10] Schreiver I, Hutzler C, Laux P, Berlien HP, Luch A (2015). Formation of highly toxic hydrogen cyanide upon ruby laser irradiation of the tattoo pigment phthalocyanine blue. Sci Rep.

[CR11] Wenzel SM, Rittmann I, Landthaler M, Bäumler W (2013). Adverse reactions after tattooing: review of the literature and comparison to results of a survey. Dermatology.

